# Integrating intention and context: assessing social cognition in adults with Asperger syndrome

**DOI:** 10.3389/fnhum.2012.00302

**Published:** 2012-11-08

**Authors:** Sandra Baez, Alexia Rattazzi, María L. Gonzalez-Gadea, Teresa Torralva, Nora Silvana Vigliecca, Jean Decety, Facundo Manes, Agustin Ibanez

**Affiliations:** ^1^Institute of Cognitive Neurology and Institute of Neuroscience, Favaloro UniversityBuenos Aires, Argentina; ^2^National Scientific and Technical Research CouncilBuenos Aires, Argentina; ^3^Pontifical Catholic University of ArgentinaBuenos Aires, Argentina; ^4^Argentinean Program for Children, Adolescents and Adults with Autism Spectrum Disorders (PANAACEA)Buenos Aires, Argentina; ^5^Research Centre of the Faculty of Philosophy and Humanities, National University of CórdobaCórdoba, Argentina; ^6^Departments of Psychology and Psychiatry, and Center for Cognitive and Social Neuroscience, University of ChicagoChicago, IL, USA; ^7^Laboratory of Cognitive Neuroscience, Universidad Diego PortalesSantiago, Chile

**Keywords:** Asperger syndrome, contextual social cognition, executive functions, individual variability

## Abstract

Deficits in social cognition are an evident clinical feature of the Asperger syndrome (AS). Although many daily life problems of adults with AS are related to social cognition impairments, few studies have conducted comprehensive research in this area. The current study examined multiple domains of social cognition in adults with AS assessing the executive functions (EF) and exploring the intra and inter-individual variability. Fifteen adult's diagnosed with AS and 15 matched healthy controls completed a battery of social cognition tasks. This battery included measures of emotion recognition, theory of mind (ToM), empathy, moral judgment, social norms knowledge, and self-monitoring behavior in social settings. We controlled for the effect of EF and explored the individual variability. The results indicated that adults with AS had a fundamental deficit in several domains of social cognition. We also found high variability in the social cognition tasks. In these tasks, AS participants obtained mostly subnormal performance. EF did not seem to play a major role in the social cognition impairments. Our results suggest that adults with AS present a pattern of social cognition deficits characterized by the decreased ability to implicitly encode and integrate contextual information in order to access to the social meaning. Nevertheless, when social information is explicitly presented or the situation can be navigated with abstract rules, performance is improved. Our findings have implications for the diagnosis and treatment of individuals with AS as well as for the neurocognitive models of this syndrome.

## Introduction

Social cognition refers to specific information processing involved in the successful navigation of challenges related to survival and reproduction in social species (Adolphs, 1999). The construct of social cognition involves several domains, including emotional processing, theory of mind (ToM), decision-making, empathy, moral judgment, and social norms knowledge, among others. Despite the seemingly differences in these components, some of them require similar underlying processes. Multiple social cognition domains require the spontaneous perception of the relevant social elements of the situation and the interpretation of how these elements create a given social context (Klin, 2000), which depends on the implicit inference of contextual clues that bias the social meaning of an action (Ibañez and Manes, 2012). For example, emotional recognition of a face usually occurs within a background that includes emotional body language and other convergent information such as prosody, gestures, and situational clues. In contrast, other processes may require the use of explicit and abstract rules about the general social setting in terms of conventions or expected behaviors (e.g., explicit social norms during specific social interactions). Thus, different strategies underlie the different social cognition domains. Here, we investigate different aspects of social cognition in adults with Asperger syndrome (AS).

AS is a pervasive developmental disorder characterized by severe and sustained impairments in social interaction and the development of restricted, repetitive patterns of behavior, interest, and activities. These disturbances must cause significant impairments in social, occupational, or other important areas of functioning (American Psychiatric Association, 1994; Matson and Wilkins, 2008). AS may be distinguished from autistic disorder by a lack of delay in early language development (Baron-Cohen et al., 2005). Because the main focus has been on early recognition and diagnosis, this syndrome has primarily been studied in children. However, given that AS is a chronic lifelong condition and nuclear symptoms persist, research in adults has recently received particular attention (Fombonne and Tidmarsh, 2003; Lugnegard et al., 2011).

Recent reports suggest that adults with AS exhibit deficits in multiple social cognition domains including face recognition, emotional processing, ToM, empathy, and moral judgment (see below). Nevertheless, previous studies have not taken into account several factors that should be considered simultaneously in the social cognition research of these individuals. These factors include: (1) the simultaneous assessment of multiple social cognition domains, (2) the sample selection, (3) the assessment of executive functions (EF), and (4) the cognitive heterogeneity of the AS. In the present study, we considered all of these aspects, which are essential for establishing the underlying factors that contribute to the social cognition deficits of adults with AS.

### Social cognition disturbances in adults with AS

Emotional processing is an emerging topic of interest. There are numerous reports of individuals with autism spectrum disorders [autism, high functioning autism (HFA), and AS] being impaired in both recognition (Hobson et al., 1988; Ashwin et al., 2006; Hubert et al., 2007; Atkinson, 2009) and production of emotional expressions (Macdonald et al., 1989). Studies focused in adults with AS (Philip et al., 2010) show deficits on emotion recognition from faces [especially negative emotions (Ashwin et al., 2007; Falkmer et al., 2011)]. Thus, evidence suggests that emotional processing is affected in AS and other autism spectrum disorders.

ToM is another area of interest in AS research, since it requires the ability to infer the beliefs, intentions, and emotions of others (Baron-Cohen et al., 1985). Adults with AS have difficulty understanding the intentions (cognitive ToM) and emotional impact of others' actions (affective ToM) as assessed by the Faux Pas Test (FPT) (Zalla et al., 2009). However, reports of adults with AS with the reading the mind in the eyes test (RMET) have shown impaired (Baron-Cohen et al., 1997, 2001) and preserved performance (Roeyers et al., 2001; Ponnet et al., 2004; Spek et al., 2010). These controversial results have been explained by the features of the RMET since correlations between RMET and other ToM measures are weak (Luzzatti et al., 2002; Spek et al., 2010).

Impairments in empathy, the capacity to share and understand the emotional states of others in reference to oneself (Decety and Moriguchi, 2007), are also a feature of the AS. Nevertheless, few studies have examined empathy in adults with AS. The majority of the studies (Baron-Cohen and Wheelwright, 2004; Rogers et al., 2007) have focused on self-report questionnaires. However, other reports (Dziobek et al., 2008) have represented an experimental assessment of empathy in adults with AS. These studies show that these patients are impaired in cognitive empathy but do not differ from controls in emotional empathy.

Finally, one study recently reported that participants with AS and HFA participants exhibit specific impairments in moral judgment. Participants made atypical moral judgments when they needed to consider the intention of harm (accidental vs. intentional) and the outcome (neutral vs. negative) of a person's actions (Moran et al., 2011). These participants were unable to judge the moral difference between accidental and attempted harms.

### Relevant factors in AS social cognition research

As we mentioned above, to establish the underlying factors that contribute to the social cognition deficits of adults with AS, it is essential to consider several factors. First, to explore the social cognitive deficits in adults with AS, it is important to examine multiple domains with different tasks. Implicit social cognition tasks would require the spontaneous perception of the relevant contextual elements of the situation (Klin, 2000). Conversely, in explicit social cognition tasks the elements of the situation are clearly defined and these can usually be solved with relatively abstract and universal rules learned by explicit knowledge. Individuals with AS fail when they need to spontaneously apply social reasoning abilities to solve more naturalistic tasks, but when explicit information is provided, they improve the performance (Klin, 2000; Senju et al., 2009; Izuma et al., 2011). Thus, to assess several social cognition domains with different contextual clues involvement allows for a more comprehensive evaluation, and it makes it possible to establish whether there is a common factor that explains the adults with AS social cognition deficits. However, until now, only a few studies have simultaneously tested more than one social cognition domain.

Furthermore, most of previous social cognition reports (Baron-Cohen et al., 2001; Baron-Cohen and Wheelwright, 2004; Moran et al., 2011; Zalla et al., 2011) have included subjects diagnosed with AS and patients with other autism spectrum disorders (e.g., HFA). Therefore, the findings of these investigations can be biased by the sample selection. There is an ongoing debate about the differentiation among autistic subtypes, especially between AS and HFA. According to the DSM-IV criteria (American Psychiatric Association, 1994) for autism, not for AS, delay in language and qualitative impairments in communication must be evident. However, several studies suggest that there is not only a difference in language abilities among HFA and AS (for a review see Matson and Wilkins, 2008). Unlike HFA, individuals with AS do not have delay in early cognitive functioning (Frith, 2004). Furthermore, AS compared to HFA individuals have more accentuated visual-motor deficits (Klin et al., 1995; Noterdaeme et al., 2010), less strong impairments in verbal comprehension (Noterdaeme et al., 2010; Planche and Lemonnier, 2012), higher verbal than performance IQ (Klin et al., 1995) and less severe behavioral abnormalities (Gilchrist et al., 2001). These evidences suggest that both of these disorders should be studied as separate diagnostic entities (Matson and Wilkins, 2008).

On the other hand, EF are required for the processing of emotional stimuli and social cognition tasks (Pessoa, 2008; Uekermann et al., 2010). Emotional processing requires holding stimuli in the working memory, and irrelevant information needs to be inhibited. In the same vein, ToM and empathy entail holding information in the working memory and switching between one's own perspective and that of another person (Uekermann et al., 2010). Nevertheless, no studies on adults with AS have controlled for the effect of EF on social cognition performance.

Finally, adults with AS perform variably among multiple domains (Hill and Bird, 2006; Towgood et al., 2009). This variability is observed more frequently in EF but also in social cognition. Deficits in working memory, cognitive flexibility and inhibitory control have been reported (Morris et al., 1999; Ambery et al., 2006; Hill and Bird, 2006), while other studies in adults with AS (Just et al., 2007; Nyden et al., 2010) have found preserved executive functioning. Affected (Baron-Cohen et al., 1997; Zalla et al., 2009) and intact performances (Ponnet et al., 2004; Spek et al., 2011) on ToM tasks have also been reported. These mixed findings suggest that patterns of deficits vary from individual to individual and that the adults with AS population include patients with both sub-normal and supra-normal performance. Thus, AS is more likely to be associated with a complex pattern of deficits across and within domains rather than just a single primary processing deficit (Happe et al., 2006). The heterogeneity in AS individuals has been interpreted as an obstacle to research (Happe et al., 2006). Traditional group-study type of analysis is problematic for individuals with high variability in performance because of the *averaging artifact* (Shallice and Evans, 1978).

### The goal of this study

The primary goal of this study was to examine the performance of adults with AS on multiple social cognition domains with different levels of contextual integration while assessing the influence of EF. The social cognition domains evaluated were emotion recognition, ToM, empathy, moral judgment, social norms knowledge, and self-monitoring behavior in social settings. We included some tasks that require the implicit perception and integration of the relevant social elements to solve a social situation, and other in which the elements of the situation are explicitly defined and can be solved with relatively abstract and universal learned rules. In adittion, we explored the individual variability in the AS group. For this purpose, we employed a methodology called *multiple case series analysis* (MCSA) (Hill and Bird, 2006; Towgood et al., 2009), that detects the domains in which a given individual displays an abnormal performance. Group comparison analyses requires homogeneity between subjects; however, individuals with AS exhibit performance variability, which is concealed in these analyses. Therefore, the lack of significant differences is not necessarily an index of intact performance in this population (Hill and Bird, 2006).

Taking previous findings into account, we predicted that adults with AS will have deficits in several social cognition domains. We hypothesized that the social cognition deficits of adults with AS would be more related to impairments in the capacity to implicitly integrate action intentions with contextual clues than to the inability to apply explicit social rules. We also hypothesized that the social cognition difficulties would not be explained by EF profiles. This hypothesis was based on the fact that deficits in social cognition seem to be a fundamental characteristic that is less affected by AS heterogeneity, while patterns of EF have shown high variability between individuals. Finally, we predicted that the MCSA should demonstrate that patterns of cognitive strengths and weaknesses vary within individuals.

## Materials and methods

### Participants

Fifteen adult's diagnosed with AS and 15 healthy subjects participated in the present study. All participants were selected from the outpatient population of the Institute of Cognitive Neurology. All adults with AS had an estimated IQ above 94 (SD ≤ 7.42). Patients were assessed by a psychiatrist and met the diagnostic and statistical manual of mental disorders (DSM-IV) criteria for AS (American Psychiatric Association, 1994). The diagnosis was made on the basis of the adult Asperger assessment (AAA) (Baron-Cohen et al., 2005). Before the clinical interview, patients are asked to complete autism spectrum quotient (AQ) and the empathy quotient (EQ) as screening questionnaires (see Table 2). The psychiatrist then sought to validate the symptom examples provided by the AQ and EQ and checked the other AS symptoms and criteria.

Healthy control participants matched with the adults with AS were recruited from a large pool of volunteers. No significant differences in age [*F*_(1, 28)_ = 0.003, *p* = 0.95], gender [*X*^2^_(1)_ = 0.012, *p* = 0.91], handedness [*X*^2^_(1)_ = 0.00, *p* = 1.00] or years of formal education [*F*_(1, 28)_ = 1.36, *p* = 0.25] were observed between adults with AS and controls.

The following exclusion criteria were applied: (1) AS participants who met DSM-IV criteria for any axis-I diagnosis were excluded; (2) control subjects with a history of mental retardation, neurological disease, psychiatric disease, or any clinical condition that may affect cognitive performance were excluded; (3) adults with AS and controls with a history of drug or alcohol abuse were also excluded. All participants provided written informed consent in agreement with the Helsinki declaration. The study was approved by the ethics committee of Institute of Cognitive Neurology.

### Materials and procedure

A battery of neuropsychological tests was used to assess EF and social cognition (see below). Patients were also evaluated with the Wechsler abbreviated scale of intelligence (WASI). This scale includes vocabulary and matrix reasoning subtests and provides an estimated IQ (Weschler, 1999). All participants were individually evaluated in a quiet office of the Institute of Cognitive Neurology. A complete evaluation was administrated in one session that lasted approximately 2 h. Subjects were initially assessed with the social cognition tasks and then with the EF and intellectual level tests. The order of administration of the tasks was the same for each participant.

#### EF assessment

All participants were evaluated with an EF battery which included measures of verbal fluency, inhibitory control, interference control, working memory, and cognitive flexibility. Verbal and design fluency tests (Delis and Kaplan, 2001) were used to assess recall, self-monitoring, and cognitive flexibility strategies. The trail-making test (Partington, 1949) was employed to assess cognitive flexibility and processing speed, and the Hayling test (Burgess and Shallice, 1996) was used to measure inhibitory control. The Flanker test (Eriksen and Eriksen, 1974) was applied to evaluate the ability to inhibit responses to irrelevant stimuli and the executive control of attention. The set shifting task (Diamond and Kirkham, 2005) was used to assess cognitive flexibility and inhibitory control. Finally, a span counting task (Case et al., 1982) and the 1-back test (Gevins and Cutillo, 1993) were applied to evaluate working memory.

#### Measures of social cognition

A description of social cognition tasks is provided in Table 1. All participants were evaluated with a social cognition battery that included measures of emotion recognition, ToM, empathy, moral judgment, social norms knowledge, and self-monitoring behavior in social settings. The awareness of social inference test (TASIT) (McDonald et al., 2003, 2006; Kipps et al., 2009) was used to assess recognition of emotional states. This task introduces contextual cues (e.g., prosody, facial movement, and gestures) and additional processing demands (e.g., adequate speed of information processing, selective attention, and social reasoning) that are not taxed when viewing static displays. The RMET (Baron-Cohen et al., 1997) and the FPT (Stone et al., 1998) were applied to assess emotional and cognitive aspects of the ToM. An empathy for pain task (EPT; Couto et al., 2012) was employed to evaluate the empathy in the context of intentional and accidental harms. We also used the interpersonal reactivity index (IRI; Davis, 1983), a 28-item self-report questionnaire that measures both the cognitive and affective components of empathy. Finally, we included a moral judgment task (Young et al., 2010) and the revised self-monitoring scale (RSMS) (Lennox and Wolfe, 1984). A detailed description of the social cognition tasks is provided in supplementary data.

**Table 1:** **Social cognition domain assessed and tasks employed**.

**Social cognition domain**	**Task**	**Description**
Emotional processing	TASIT	This task assesses recognition of emotional expressions. The test introduces contextual cues and additional processing demands that are not taxed when viewing static displays.
Theory of mind	RMET	This test assesses the emotional inference aspect of the ToM. Consist of 17 pictures of the eye region of a face. Participants are asked to choose which of four words best describes what the person in each photograph is thinking or feeling.
	FPT	The FPT assesses the emotional and cognitive inference aspects of the ToM. In this task, the participants read stories that may contain a social faux pas. The subject is asked whether someone said something awkward. Performance was scored regarding the adequate identification of the faux pas (hits) and the adequate rejection of those stories which did not contain a faux pas (rejects). A total score was computed by adding the number of hits and rejects. When a faux pas was correctly identified, subjects were also asked 2 additional questions to measure intentionality—that is, recognizing that the person committing the faux pas was unaware that they had said something inappropriate—and emotional attribution, in which participants should recognize that the person hearing the faux pas might have felt hurt or insulted.
Empathy	EPT	This task evaluates the empathy in the context of intentional and accidental harms. Consists of 25 animated situations involving two individuals that are presented successively. The three following kinds of situations were depicted: intentional pain in which one person is in a painful situation caused intentionally by another; accidental pain where one person is in a painful situation accidentally caused by another; and control or neutral situations. We assessed 7 questions about the following aspects of the scenarios: intentionality; emphatic concern; degree of discomfort; harmful behavior; the valence behavior of the active perpetrator; the correctness of the action, and finally punishment (see Appendix for a detailed description). Each question was answered using a computer-based visual analog scale giving 7 different ratings by trial. Accuracy, reaction times (RTs) and ratings were measured.
	IRI	The IRI is a 28-item self-report questionnaire that separately measures both the cognitive and affective components of empathy.
Moral judgment	Moral judgment task	We presented participants with 24 scenarios with four possible variations following a 2 × 2 design: (1) the protagonists either harmed another person (negative outcome) or did no harm (neutral outcome); (2) the protagonists either believed that they would cause harm (negative intent) or believed that they would cause no harm (neutral intent). Participants were asked to rate the scenario on a Likert scale ranging from totally permissible (7) to totally forbidden (1).
Social norms knowledge	SNQ	The SNQ questionnaire consisting of 20 yes–no questions. The participants were asked to determine whether a behavior would be appropriate in the presence of an acquaintance according to the mainstream culture.
Self-monitoring behavior in social settings	RSMS	The RSMS is a 13-item instrument and assesses the tendency to regulate one's behavior to present a particular self in a social context. The scale involves two styles of self-monitoring behavior: the ability to modify self-presentation and the sensitivity to the expressive behavior of others.

TASIT, The awareness of social inference test; RMET, Reading the mind in the eyes test; FPT, Faux Pas test; EPT, empathy for pain task; IRI, index of interpersonal reactivity; SNQ, social norms questionnaire; RSMS, revised self-monitoring scale.

### Data analysis

The demographic, neuropsychological, and experimental data were compared between the groups using ANOVA and Tukey's HSD *post-hoc* test (when appropriate). The ANOVA results were also corrected for multiple comparisons using the Tukey's test. When analyzing categorical variables (e.g., gender), χ ^2^ square tests were applied. To control for the influence of EF on the performance on social cognition tasks, we applied an ANCOVA test that was adjusted for the cognitive flexibility score. The α value for all statistical tests was set at 0.05.

To assess individual differences, we conducted a MCSA and compared each participant with the control group on every performance measure. We followed the method of Towgood et al. (2009) and used a threshold of 2 standard deviations (SD) from the mean of the control group to define the normal range. First, we identified control subjects who displayed abnormal performance in each sub-measure, according to the 2 SD criteria, and removed them. Then, we recomputed the control means and SD excluding these subjects and identified adults with AS and control participants who were below (minus 2 SD) or above (plus 2 SD) the controls mean. We carried out frequency analyses in order to record the instances in which the performance of each subject was subnormal or supranormal. We then used non-parametric tests (Mann–Whitney tests) to compare the number of measures of impaired and supra-normal performance.

Finally, Pearson's correlations were performed to examine the association between the EF measures with the greatest variability, and the total scores on the social cognition tasks that were significantly different between groups.

## Results

Table 2 shows the overall results from the demographic and EF assessment.

**Table 2:** **Demographic and executive functions assessment**.

	**AS (*n* = 15)**	**Control (*n* = 15)**	***P***
**DEMOGRAPHICS**			
Age (years)	35.46 (11.86)	35.7 (11.52)	0.95
Gender (M:F)	11:4	11:4	0.91
Education (years)	15.33 (3.55)	16.66 (2.60)	0.25
WAT	39.21 (4.09)	39.07 (4.81)	0.93
Handedness (L:R)	0:15	0:15	1.00
Autism Spectrum Quotient	34.14 (6.17)	−	−
Empathy Quotient	18.57 (10.53)	−	−
**EXECUTIVE FUNCTIONS**			
Phonological fluency	13.10 (4.78)	14.92 (2.43)	0.21
Simple design fluency	8.50 (2.71)	10.00 (3.01)	0.17
Switching design fluency	8.9 (2.70)	11.14 (2.47)	**0.03**
T.M.T-B	74.50 (27.23)	63.30 (14.17)	0.19
Hayling Test	9.07 (7.36)	6.00 (3.89)	0.19
*Flanker Task*			
Reaction Time (congruent)	667.32 (164.66)	629.11 (134.89)	0.52
Accuracy (congruent)	99.71 (0.89)	99.77 (0.83)	0.85
Reaction Time (incongruent)	718.50 (145.11)	713.19 (121.49)	0.91
Accuracy (incongruent)	98.57(2.45)	98.65 (2.02)	0.92
*Set Shifting Task*			
Reaction Time (shape)	602.15 (118.08)	632.04 (191.65)	0.43
Accuracy (shape)	93.74(2.78)	97.91 (3.56)	0.38
Reaction Time (color)	654.78 (314.38)	588.42 (130.57)	0.47
Accuracy (color)	97.91 (4.27)	98.47 (2.12)	0.67
Reaction Time (incongruent)	794.03 (166.71)	745.03 (192.72)	0.24
Accuracy (incongruent)	95.82 (2.84)	96.42 (2.65)	0.59
*1-Back*			
Reaction Time	870.58 (169.03)	825.90 (173.86)	0.50
Accuracy	90.24 (12.59)	88.80 (8.53)	0.72
Dot counting task	23.92 (11.16)	24.28 (11.11)	0.93

Note: The results are shown as the mean (SD). Statistical results are shown in the right column. Significant differences are in bold.

TMT, Trail Making Test; WAT, Word accentuation test.

### Executive functions assessment

The results showed that our groups have similar EF performance. No differences in verbal fluency, inhibitory control, interference control, or working memory were observed (Table 2). However, the adults with AS performed significantly lower than controls on the switching design fluency task [*F*_(1, 28)_ = 5.10, *p* < 0.05], suggesting subtle cognitive flexibility impairments. Given these results, we considered this measure as a covariate in the social cognition performance analysis.

### Measures of social cognition

**Figure 1:**
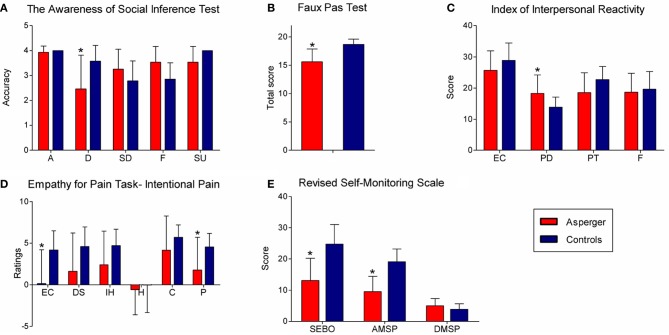
**Significant differences between groups in social cognition tasks. (A)** TASIT (accuracy per category). A, anger; D, disgust; SD, sadness; F, fear; SR, surprise. **(B)** Faux pas test (total score). **(C)** Scores on IRI subscales. EC, empathic concern; PD, personal distress; PT, perspective taking; F, fantasy. **(D)** Empathy for pain task, ratings for intentional pain situations. EC, empathic concern; DS, discomfort; IH, intention to hurt; H, happiness; C, correctness; P, punishment. **(E)** Scores on RSMS subscales. SEBO, sensitivity for expression behavior of others; AMSP, ability to modify self-presentation; DMSP, difficulty to modify self-presentation.

Figure 1 summarizes the significant differences between groups.

#### Recognition of emotional states

No significant differences in the TASIT total score were observed [*F*_(1, 28)_ = 0.69, *p* = 0.41]. The per category analysis showed significant differences between groups [*F*_(4, 108)_ = 7.97, *p* < 0.01]. A *post-hoc* analysis (Tukey HSD, *MS* = 0.49, *df* = 134.13) revealed that adults with AS had difficulty with disgust categorization (*p* < 0.01). This effect was preserved (*p* < 0.01) after co-varying for cognitive flexibility (*p* = 0.35). No significant differences were observed for anger (*p* = 1), fear (*p* = 0.22), sadness (*p* = 0.11) or surprise (*p* = 0.74) categorization.

#### Theory of mind

For the ToM measures, the adults with AS scored significantly lower than controls on the FPT total score [*F*_(1, 28)_ = 20.62, *p* < 0.01]. This result did not change (*p* < 0.01) after adjusting for cognitive flexibility (*p* = 0.15). Significant differences were also observed on the hits [*F*_(1, 28)_ = 20.62, *p* < 0.01]. Differences were preserved (*p* < 0.01) after co-varying for cognitive flexibility (*p* = 0.13). The AS group also showed lower intentionality scores [*F*_(1, 28)_ = 74.21, *p* < 0.01]. This effect was preserved (*p* < 0.01) in the covariate analysis (*p* = 0.41). Furthermore, adults with AS scored lower on emotional attribution [*F*_(1, 28)_ = 29.08, *p* < 0.01]. This effect was maintained (*p* < 0.01) after adjusting for the covariate (*p* = 0.43). No significant differences were observed on the reject scores [*F*_(1, 28)_ = 0.007, *p* = 0.93].

No differences between the groups were observed on the RMET [*F*_(1, 28)_ = 0.09, *p* = 0.76].

#### Empathy

***Empathy for pain task***. The ratings of empathic concern were significantly different between groups [*F*_(2, 52)_ = 6.70, *p* < 0.01]. A *post-hoc* analysis (tukey hsd, *ms* = 10.62, *df* = 55.08) revealed that the adults with as rated the intentional pain situations with lower scores (*p* < 0.01), even controlling for cognitive flexibility (*p* = 0.65). Furthermore, the controls rated greater empathic concern for intentional harm situations than accidental harm situations (*p* < 0.01). However, this difference was not observed in the adults with as. Moreover, significant group differences were observed in the punishment ratings [*F*_(2, 52)_ = 7.02, *p* < 0.01]. The *post-hoc* comparisons (tukey hsd, *ms* = 6.87, *df* = 66.7) showed that the adults with as tended to rate intentional harm situations with lower scores than controls (*p* = 0.06). This tendency did not change (*p* = 0.06) in the covariate analysis (*p* = 0.93). No differences were observed in the judgments of discomfort, intention to harm or correctness.

In addition, the RTs of the discomfort judgments were different between groups [*F*_(2, 52)_ = 4.72, *p* < 0.05]. The RTs of the discomfort judgments were longer for the intentional harm than the neutral (*p* < 0.01) and accidental (*p* < 0.05) harm situations. These differences were preserved (*p* < 0.05) in the covariate analysis (*p* = 0.17).

***IRI***. Adults with as scored higher on pd subscale [*F*_(1, 28)_ = 6.02, *p* < 0.05] than controls. This effect was preserved (*p* < 0.05) after adjusting for the covariate (*p* = 0.60). No difference between groups [*F*_(1, 28)_ =1.96, *p* = 0.17] were observed on the ec subscale. Furthermore, the as group tended to have lower scores than controls [*F*_(1, 28)_ =4.01, *p* = 0.055] on the pt subscale. This tendency was true (*p* < 0.01) after controlling for cognitive flexibility (*p* = 0.09). No difference between the groups was observed [*F*_(1, 28)_ = 0.17, *p* = 0.67] on the *F*-subscale.

#### Moral judgment

In both groups, actions with neutral intentions [*F*_(1, 28)_ = 146.29, *p* < 0.01] and neutral outcomes [*F*_(1, 28)_ = 24.55, *p* < 0.01] were judged to be more permissible than actions with negative intentions and negative outcomes. Accidental harm was judged as being more permissible than intentional harm (Intention × Outcome Interaction) [*F*_(1, 28)_ = 7.40, *p* < 0.01]. The group × intention × outcome interaction [*F*_(1, 28)_ = 1.60, *p* = 0.21] was not statistically significant. Therefore, the adults with AS and controls did not differ in their judgments of morality. Specifically, the judgments of the neutral (neutral outcome, neutral intent), attempted harm (neutral outcome, harmful intent), accidental harm (harmful outcome, neutral intent), or intentional harm (harmful outcome, harmful intent) vignettes did not differ between groups.

#### Knowledge of social norms

No differences between groups were observed in the break [*F*_(1, 24)_ = 0.50, *p* = 0.48] and over-adhere [*F*_(1, 28)_ = 0.00, *p* = 1.00] scores of the SNQ.

#### Self-monitoring behavior in social settings

Adults with AS obtained lower scores in the sensitivity for expression behavior of others compared to controls [*F*_(1, 28)_ = 29.26 *p* < 0.01], even after the covariate (*p* = 0.65). Adults with AS also received lower scores on the ability to modify self-presentation [*F*_(1, 28)_ = 19.40, *p* < 0.01]. This effect remained true after the covariate analysis (*p* < 0.01), even though a significant effect of cognitive flexibility (*p* < 0.05) on self-presentation was observed.

In summary, adults with AS showed impairments on measures of disgust recognition (TASIT), ToM (FPT), and empathic concern and punishment ratings for the intentional harm situations (EPT). Additionally, the adults with AS showed higher scores on the PD subscale. They also showed lower scores on subscales of the sensitivity to the expressive behavior of others and the ability to modify self-presentation (RSMS). All differences were preserved after covarying for cognitive flexibility. Overall, adults with AS seem to perform less well in tasks that require an implicit encoding of socially relevant information and automatic context integration. Nevertheless, they performed as well as controls in tasks in which the social information was explicitly presented and when the task could be solved with abstract rules. Finally, the difficulties experienced by the adults with AS were not explained by abnormalities in EF.

### Multiple case series analysis (MCSA)

To explore the intra-individual variability in tasks performance of the AS group, we examined the ranges of *z*-scores based on the performance of the control group (Towgood et al., 2009). The maximum range of performance on each of the 78 measures in controls was 4.60. Among the adults with AS, more than 43% of the measures (34/78 sub-measures) showed a *z*-score range exceeding the maximum threshold observed in controls. Specifically, 27.78% (5/18) of the EF measures exceeded the maximum range of the control group, whereas 48.33% (29/60) of the social cognition measures exceeded this range.

A greater number of adults with AS performed atypically compared with the control group. The individual performance profiles of each AS and control participants are provided in Appendix (see Tables A1a, A1b, A2a and A2b). The measures that were the most variable are detailed in Table 3. Most of the adults with AS performed below normal (<2SD below control group mean) in both, EF and social cognition measures. With regard to EF, supra-normal (>2SD above control group mean) performance was observed only in the phonological fluency task. They also obtained supra-normal performance on several EPT measures. More specifically, the adults with AS showed supra-normal ratings in tasks involving neutral situations (e.g., discomfort, intention to hurt, and happiness ratings). In neutral scenarios in which the actions do not involve the intention to hurting someone, one would expect lower discomfort or intention to hurt ratings. Thus, the results suggest that the adults with AS are unable to discriminate between the neutral, accidental and intentional pain situations.

**Table 3:** **The measures of executive functions and social cognition reveal variable performance in the AS group**.

**Measure**	**Range (*z*-scores)**	**>2SDs (%)**	**<2SDs (%)**
**EXECUTIVE FUNCTIONS MEASURES**			
Phonological fluency	8.40	13.33	33.33
Hayling Test	6.68	0	26.67
Trail Making Test-B	6.07	0	26.67
Set Shifting accuracy (color)	5.87	0	13.33
1-Back accuracy	5.08	0	6.66
**SOCIAL COGNITION MEASURES**			
Faux Pas Test	7.39	0	60
The Awareness of Social Inference Test	6.0	0	20
Over adhere score—Social Norms Questionnaire	6.86	0	13.33
Break score—Social Norms Questionnaire	6.22	0	6.66
*Empathy Task for Pain*			
Situation comprehension RT (neutral situations)	7.71	0	20
Intentionality rating (neutral situations)	11.23	0	20
Intentionality rating (intentional pain)	10.96	0	13.33
Intentionality rating (accidental pain)	8.05	0	33.33
Emphatic concern rating (intentional pain)	5.54	0	33.33
Emphatic concern rating (accidental pain)	5.69	0	6.66
Discomfort rating (neutral situations)	5.67	20	0
Discomfort rating (intentional pain)	6.76	0	33.33
Discomfort RT (intentional pain)	7.52	0	26.67
Discomfort RT (accidental pain)	6.45	0	6.66
Intention to hurt rating (neutral situations)	5.52	20	0
Intention to hurt RT (neutral situations)	4.61	0	6.66
Intention to hurt rating (intentional pain)	8.20	0	26.67
Happiness rating (accidental pain)	4.76	20	0
Happiness RT (neutral situations)	5.70	0	13.33
Correctness rating (neutral situations)	7.40	26.67	0
Correctness RT (neutral situations)	4.82	0	6.66
Correctness rating (intentional pain)	11.09	0	20
Correctness RT (accidental pain)	5.79	0	13.33
Punishment rating (neutral situations)	9.49	33.33	0
Punishment RT (neutral situations)	6.21	0	33.33
Punishment rating (intentional pain)	9.96	6.66	33.33
Punishment RT (intentional pain)	4.96	0	13.33
Punishment RT (accidental pain)	4.99	0	13.33

Consistent with the group analysis, the MCSA revealed that the adults with AS performed less well than the controls. Inter-individual variability (subnormal performance) was observed on: FPT (60%), TASIT (26.67%), empathic concern rating of intentional pain (33.33%), PD (33.33%), sensitivity of expression behavior of others (33.33%) and ability to modify self-presentation (53.33%).

To explore the inter-individual variability, we analyzed the performance of each participant and recorded instances in which the performance was 2 SDs below or above of the control mean. A non-parametric test was applied to compare the number of measures for subnormal and supra-normal performance (see Table 4). As expected, the adults with AS showed a greater number of abnormal measures than controls (Mann–Whitney *U* = 19.00, *p* < 0.01). The AS participants also showed a greater number of measures in which they performed below control performance (Mann–Whitney *U* = 14.00, *p* < 0.01). However, no significant differences were observed in the number of measures with supra-normal performance (Mann–Whitney *U* = 82.00, *p* = 0.21).

**Table 4:** **Comparison of the number of measures in which each individual exhibited abnormal performance**.

	**Median**	**SD**	**Range**	***p***
Controls: measures supra-normal	0.80	2.41	0-9	0.21
AS: measures supra-normal	2.13	2.55	0-8	−
Controls: measures subnormal	1.60	1.08	0-3	0.000007
AS: measures subnormal	10.33	6.87	1-29	−
Controls: total measures abnormal	2.40	2.87	0-10	0.000027

In summary, the MCSA showed higher variability in the performance of the adults with AS compared with controls. A larger proportion of the social cognition measures compared to the EF measures exceeded the maximum range of the *z*-scores calculated based on the control group performance. In the AS group subnormal performance was higher than supra-normal.

### Association between EF and social cognition performance

Finally, we explored the influence of EF on social cognition performance. We examined the correlation between the EF measures with the greatest variability, and the total scores on the social cognition tasks that were significantly different between groups. No significant correlations were observed.

## Discussion

The primary goal of this study was to examine the performance of adults with AS on tasks of multiple domains of social cognition, while assessing the influence of EF. The secondary goal was to explore individual variability in adults with AS performance on both the social cognition and EF tasks. Our results suggest that participants with AS have a fundamental deficit in several domains of social cognition. We also found that the AS participants showed a greater number of social cognition measures in which they performed below controls' performance. These deficits were not explained by abnormalities in EF.

Furthermore, our data suggest that a common mechanism underlies the deficits in multiple social cognition domains in the adults with AS. In brief, these participants performed poorly on tasks (TASIT, FPT, EPT) that imply the ability to implicitly infer the intentionality of actions and those that require the integration of mental states (intentions, beliefs, emotions) with contextual information.

This is the first study in adults with AS to explore the effect of EF on social cognition performance. Both AS and control groups were similar regarding executive functioning. Moreover, to control for the effect of EF on performance during social cognition tasks, we conducted covariance analysis adjusted for cognitive flexibility, the only domain in which we found group significant differences. All significant differences in the social cognition measures remained significant. Moreover, we did not find significant correlations between scores on the EF measures with higher variability and those of the social cognition tasks that were different between groups. Because we selected tasks that were designed to assess specifically EF and have been utilized extensively to assess these domains (Partington, 1949; Eriksen and Eriksen, 1974; Case et al., 1982; Gevins and Cutillo, 1993; Burgess and Shallice, 1996; Delis and Kaplan, 2001; Diamond and Kirkham, 2005), we consider that the failure to find significant correlations could not be explained by the lack of the sensitivity of the executive measures. Instead, the lack of significant correlations may be explained by the low variability observed in the EF performance, since both groups had a similar executive functioning and low variability. Consequently, these results indicate that EF do not seem to play a major role in the social cognition impairments of adults with AS.

### Deficits in social cognition

We employed an ecological task of contextual inference of emotional states (TASIT) which requires the integration of cues from face, prosody, gesture, and social context to identify the emotions. Consistent with previous reports (Ashwin et al., 2007; Falkmer et al., 2011), our results showed that individuals with AS have difficulty recognizing expressions of disgust. It has been shown that the basal ganglia, in parallel with the insula, are involved in disgust recognition (Calder et al., 2000; Adolphs, 2002; Wang et al., 2003; Ibáñez et al., 2010a,b). Fronto-insular networks seem to be crucial for social cognition (Couto et al., 2012). Individuals with AS show reduced gray matter in the basal ganglia (McAlonan et al., 2002; Nayate et al., 2005). They also show abnormalities in the white matter between the basal ganglia and thalamus, which connects brain areas (amygdala and fusiform gyrus) (McAlonan et al., 2009). Moreover, adults with AS present smaller volumes in the insular cortex (Kosaka et al., 2010). Therefore, the deficits in disgust recognition may be associated with abnormalities in the basal ganglia and the insula.

As previously reported (Ponnet et al., 2004; Spek et al., 2011), no differences between AS individuals and controls were found in ToM as measured by the RMET. Nevertheless, our data showed that the adults with AS performed poorly on the FPT, which is consistent with other studies (Zalla et al., 2009; Spek et al., 2011). In this test, adults with AS failed to identify the faux pas and to understand them as unintentional actions. Furthermore, they had difficulties to understand the emotional impact generated by the faux pas. The discrepancy in the performance between both ToM tests in the AS group can be explained by the features of these tasks. First, the FPT presents social scenarios resembling daily life situations. These tasks that involve real-life social scenarios are more sensitive to detect the ToM deficits of individuals with autism and AS (Klin, 2000). Furthermore, an adequate performance in the FPT involves the capacity to implicitly integrate cognitive inferences about mental states with empathic understanding. This capacity is mediated by the appraisal of contextual clues and relevant social elements provided in the scene information. Conversely, the RMET can be solved using basic and general matching strategies to correctly pair the depicted eyes and emotions. Thus, taken together, the ToM results suggest that adults with AS have difficulty integrating implicit information from the context and using this information to infer the intentionality and the emotional impact of the others' actions.

We employed a more ecologically valid measure of empathy (EPT) than the self-report questionnaires. In this task, the adults with AS showed abnormal empathic concern ratings, punishment ratings, and RTs of discomfort judgments for the intentional pain situations. Consistent with previous findings (Klin, 2000; Zalla et al., 2009), our results indicate that these individuals have difficulty with inferring the intentionality of actions. Information about intentionality allows us to decide how bad or good an action is. The deficit in intention inference may have affected the empathic concern ratings and therefore, the punishment ratings of the adults with AS.

In addition, the adults with AS showed higher levels of PD and a trend toward lower levels of PT compared with controls on the IRI. These results are supported by previous studies (Rogers et al., 2007; Dziobek et al., 2008). The high PD scores indicate greater levels of discomfort in interpersonal settings. This finding may be related to the slower RTs in the AS group for discomfort judgments in the intentional pain situations. Furthermore, individuals with AS show higher levels of anxiety (Hurtig et al., 2009; Lai et al., 2011), which may increase their PD scores. The lower scores on the PT subscale suggest that individuals with AS have difficulty understanding the feelings and perspectives of others, which is congruent with the EPT results.

In summary, the pattern of performance on the empathy measures indicated that adults with AS are impaired when using contextual information to infer the intentions of others. These deficits are reflected by lower ratings of empathic concern and punishment. Moreover, these individuals show higher levels of discomfort in stressful interpersonal situations.

Interestingly, we found that adults with AS performed similarly than control participants on measures of moral judgment. Both groups judged accidental harm as being more permissible than intentional harm. The lack of difference between groups in this task may be due to the fact that information about intention, outcome, and context (scene information) were presented in an explicit way. Therefore, it was possible to understand the moral content using two abstract rules with a linear relationship. For example, if the protagonist had the intention of harming another person (negative intent) and in fact caused harm (negative outcome); then the protagonist's action should be morally forbidden. Our results are in line with previous studies in individuals with AS (Klin, 2000; Izuma et al., 2011) that have shown intact performance or subtle deficits on tasks where explicit information is available. However, a recent study (Moran et al., 2011) employing a similar paradigm reported atypical moral judgment in individuals with AS and HFA. The discrepancy between these results and the current findings may be explained by the sample selection criteria employed in each study. Moran and colleagues included both HFA and AS participants. Individuals with HFA have language delay and usually present impairments in verbal skills (Baron-Cohen et al., 2005; Matson and Wilkins, 2008). These difficulties can affect their performance on the task. Thus, moral judgment in adults with AS needs to be further studied using naturalistic social situations without explicit rules.

On the other hand, this is the first attempt to investigate self-monitoring in social settings in an AS population. As expected, AS participants were less sensitive to the expressive behavior of other individuals, indicating that they had a low capacity for detecting implicit social and interpersonal cues. They also showed a diminished ability to modify self-presentation in social situations, suggesting that they had difficulty with adjusting their behaviors and with navigating novel or challenging social situations. Consistent with this idea, a negative correlation between self-monitoring and measures of social skills has been reported (Furnham and Capon, 1983). Furthermore, the ability to modify self-presentation is negatively correlated with social anxiety (Cramer and Gruman, 2002). Thus, the deficits in self-monitoring in social settings may be related to the lack of social skills and the high levels of anxiety (Hurtig et al., 2009; Lai et al., 2011) experienced by individuals with AS.

Moreover, our results revealed no differences between the AS participants and controls on the SNQ. This finding indicates that social rules knowledge is preserved in adults with AS. In accordance with our data, a study (Zalla et al., 2011) reported that AS and high-functioning individuals with autism are able to detect social rule violations. Furthermore, social norms can be learned in an explicit way. This explicit knowledge can be used by adults with AS to guide their behavior and can act as a compensatory strategy for their social cognition deficits.

Overall, consistent with our hypothesis, the adults with AS showed impairments in several social cognition domains (emotion recognition, ToM, empathy, and self-monitoring in social settings). Specifically, the adults with AS performed poorly on those social cognition tasks (TASIT, FPT, and EPT) that involve an implicit encoding of socially relevant information and the automatic integration of contextual information to solve a given social situation. Conversely, these individuals performed as well as controls in some tasks (RMET, moral judgment task, and SNQ) that had common features. In these tasks the elements of the situation are clearly defined and usually can be solved with relatively abstract and universal rules. This pattern of social cognition performance suggests that one underlying factor may explain the deficits. According to a recently proposed social context network model (Ibañez and Manes, 2012), this factor seems to be the implicit encoding and the integration of contextual information in order to access to the social meaning.

In addition, our results suggest that adults with AS may benefit from the use of explicit information. However, in most real-life situations, the social demands are not explicitly formulated. Social situations involve implicitly inferring the meaning of the circumstance by integrating contextual cues. Therefore, the pattern of deficits presented here may partially explain the difficulties with social interaction that individuals with AS experience in their daily lives.

Adults with AS may use abstract rules to compensate for their impairments in social cognition. Previous reports have shown that individuals with AS have superior abstract reasoning abilities (Hayashi et al., 2008; Soulieres et al., 2011). This strength may contribute to the performance on social cognition tasks that require the use of abstract rules and the integration of explicit information. On the other hand, this superiority in abstract reasoning may not help in social situations that involve implicit social rules and the integration of contextual cues. In these situations, the meaning of social information is less predictable and relies heavily on context, which reduces the chances of inferring the meaning by applying explicit abstract rules.

### Variability in the performance of adults with AS

Adults with AS showed heterogeneous performance on several EF and social cognition tasks. These participants obtained mainly subnormal performance among the measures with the largest variability. Furthermore, this intra-individual variability was higher for the performances of social cognition than for the EF tests. The decreased variability of the EF tasks can be explained by the intact or superior fluid intelligence in adults with AS (Hayashi et al., 2008; Soulieres et al., 2011). Fluid intelligence is a major dimension of individual differences and refers to reasoning, abstract though and novel problem-solving ability (Duncan et al., 1995; Gray et al., 2003). Previous studies have suggested that high fluid intelligence is associated with better scores on EF tasks (Gray et al., 2003; Burgess and Braver, 2010) and indirectly related to psychosocial cognition (Huepe et al., 2011).

The current study is the first to explore the intra-individual variability of social cognition measures in adults with AS. Consistent with the group analysis, these patients obtained sub-normal performance on the same tasks (TASIT, FPT, EPT, IRI, and RSMS). Our data indicates that social cognition performance of adults with AS does not follow the same pattern of strengths and weaknesses reported in other cognitive domains (Hill and Bird, 2006; Towgood et al., 2009). Conversely, the social cognition patterns of individuals with AS is characterized by sub-normal performance, suggesting that these deficits are probably the core of the disorder.

## Conclusions

Our study documents multiple social cognition deficits as fundamental features of the AS diagnosis. Our results showed that adults with AS present deficits in the implicit integration of contextual information in order to access to the social meaning. However, when social information is explicitly presented and the situation can be solved with abstract rules, the individuals with AS usually perform as well as controls. We also found that individual profiles of adults with AS showed subnormal performance in social cognition measures.

This is the first report in adults with AS to evaluate multiple social cognition domains assessing the EF and exploring inter- and intra-individual variability. However, some limitations of this study should be acknowledged. First, our sample size is relatively small, but it is similar to previous social cognition studies (Dziobek et al., 2008; Zalla et al., 2009; Moran et al., 2011) and it is also similar to other reports that have explored the cognitive variability of adults with AS (Hill and Bird, 2006; Towgood et al., 2009) and other patient populations (Deloche et al., 1999; Ramus et al., 2003). Moreover, unlike other reports (Baron-Cohen et al., 2001; Baron-Cohen and Wheelwright, 2004; Moran et al., 2011; Zalla et al., 2011), we only included individuals diagnosed with AS. Second, given the ongoing debate about the differentiation among autistic subtypes, especially between AS and HFA, future studies should compare social cognition profiles of both conditions. Further research should also explore the variability patterns of adults with AS compared with HFA. Third, although AS will probably be formally excluded as a diagnostic category in the DSM-V, our findings are still relevant for studying individual differences within autism spectrum disorders and the subset of people who show a particular profile (previously diagnosed as individuals with AS). In the future, detailed scientific assessments on cognitive domains, such as the ones presented in this work, may help to identify subcategories of autism spectrum disorders.

From a theoretical perspective, our findings are relevant for discussions on social cognition domain specificity in adults with AS. As previously proposed (Stone and Gerrans, 2006a,b), our results support a social cognition profile involving different degrees of affectation and a heterogeneous profile. These results do not support a modular or the “all or nothing” structure of social cognition. Contextual processing seems to affect the social cognition profile of adults with AS in a dissimilar way. For instance, their performance on social cognition tasks may be partially explained by the interaction of low-level mechanisms with the general capacity to integrate contextual information.

From a clinical perspective, our findings may have important implications for the diagnosis and treatment of the AS. The deficits found in multiple social cognition domains seem to be the core feature of the AS. It is also important to promote the use of tasks involving real-life social scenarios because these assessments are more sensitive to AS impairments (Klin, 2000). “Ecological” measures are context-sensitive tools that should be applied in neuropsychiatry (Burgess et al., 2009; Torralva et al., 2009; Ibañez and Manes, 2012).

In addition, the traditional social skills interventions for individuals with AS are based on learning explicit rules to build and foster relationships with others (Cappadocia and Weiss, 2011). However, the social skills acquired during those interventions do not generalize to situations outside of the treatment setting, which limits the efficacy of these programs (Rao et al., 2008; Cappadocia and Weiss, 2011). Thus, incorporating naturalistic environments into treatment may help individuals with AS generalize the learned social skills. Contextual integration of situated information seems to be crucial for several cognitive processes (Ibáñez et al., 2006, 2010a,b, 2011a,b, 2012; Hurtado et al., 2009; Aravena et al., 2010; Riveros et al., 2010; Amoruso et al., 2011, 2012; Barutta et al., 2011; Couto et al., 2012; Ibañez and Manes, 2012). Although implementation would be challenging, intervention programs should be based on teaching implicit rules for interpreting unpredictable social contexts. Learning to assess implicit contextual clues may improve the social skills of adults with AS.

### Conflict of interest statement

The authors declare that the research was conducted in the absence of any commercial or financial relationships that could be construed as a potential conflict of interest.

## Figures and Tables

**Table A1a:**
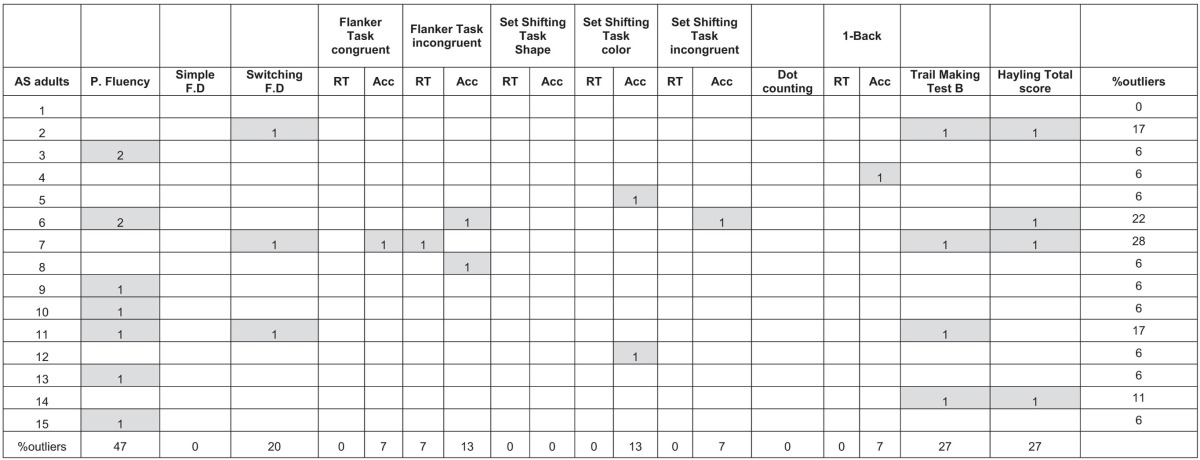
**Individual profiles of executive functions tasks performance for each adult with the AS**.

Cells numbered with “1” depict subjects whose performance was 2 SDs above the control mean (subnormal performers). Cells numbered with “2” depict subjects whose performance was 2 SDs below the control mean (supra-normal performers).

Note. P. Fluency, Phonological Fluency; Simple F.D., Simple Fluency Design Task; Switching F.D., Switching Fluency Design Task; RT, reaction Time; ACC, accuracy.

**Table A1b:**
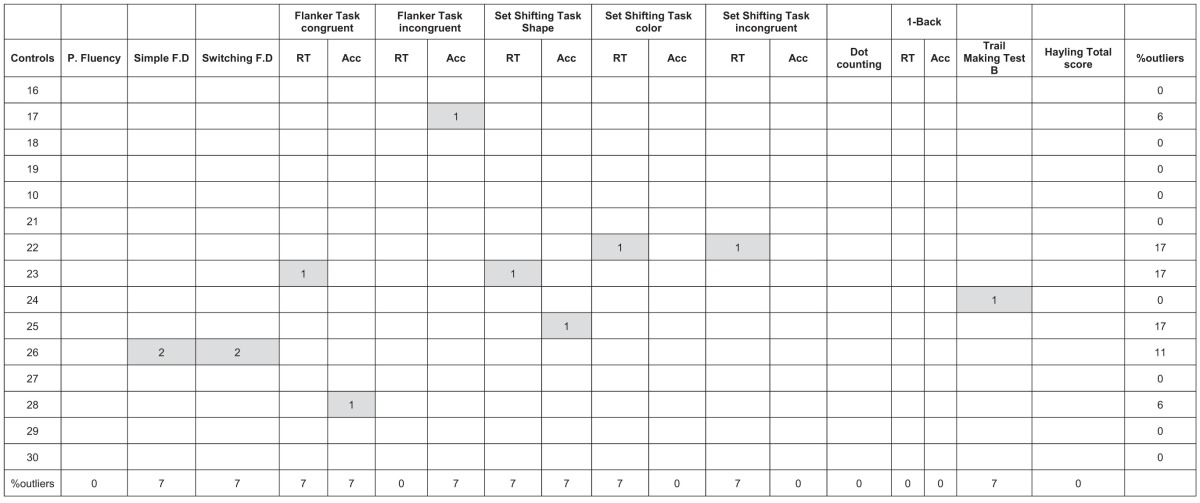
**Individual profiles of executive functions tasks performance for each control adult**.

Cells numbered with “1” depict subjects whose performance was 2 SDs above the control mean (impaired performers). Cells numbered with “2” depict subjects whose performance was 2 SDs below the control mean (supra-normal performers).

Note. P. Fluency, Phonological Fluency; Simple F.D., Simple Fluency Design Task; Switching F.D., Switching Fluency Design Task; RT, reaction Time; Acc, accuracy.

**Table A2a:**
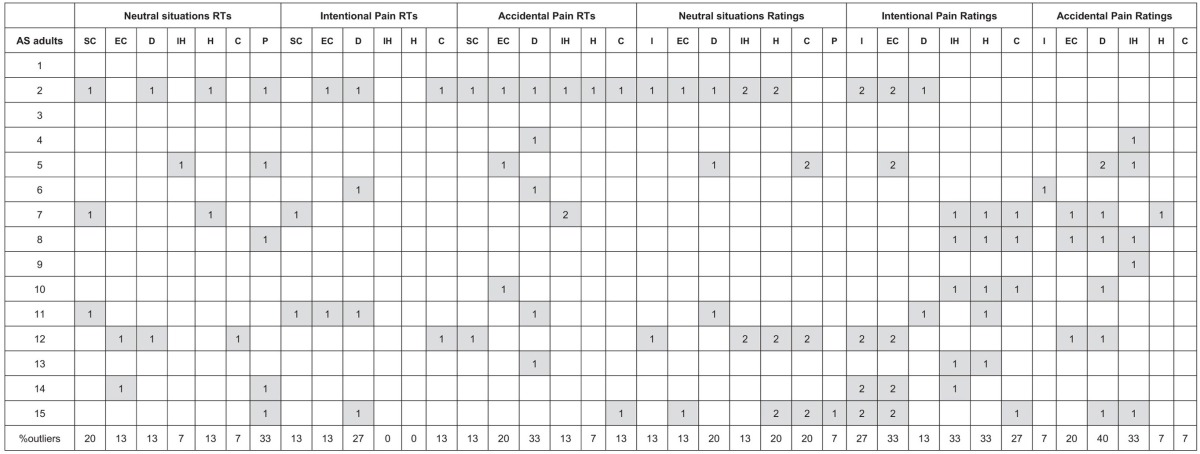
**Individual profiles of social cognition tasks performance for each adult with the AS**.

Cells numbered with “1” depict subjects whose performance was 2 SDs above the control mean (subnormal performers). Cells numbered with “2” depict subjects whose performance was 2 SDs below the control mean (supra-normal performers).

Note. RTs = reaction times; SC = situation comprehension; EC = empathic concern; D=discomfort; IH=intention to hurt; C=correctness; P=punishment; I=intentionality.

**Table A2b:**
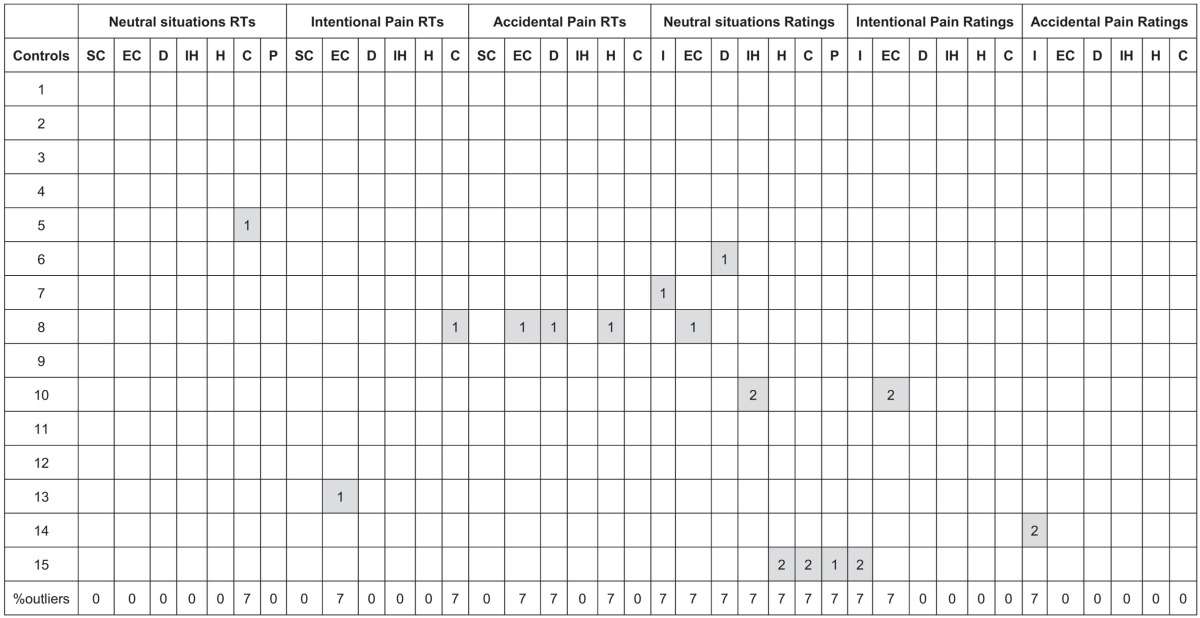
**Individual profiles of social cognition tasks performance for each control adult**.

Cells numbered with “1” depict subjects whose performance was 2 SDs above the control mean (subnormal performers). Cells numbered with “2” depict subjects whose performance was 2 SDs below the control mean (supra-normal performers).

Note. RTs, reaction times; SC, situation comprehension; EC, empathic concern; D, discomfort; IH, intention to hurt; C, correctness; P, punishment; I, intentionality.

## Appendix

### Measures of social cognition

#### Recognition of emotional states

The awareness of social inference test (TASIT). The TASIT is a test of social perception that involves videotaped vignettes of everyday social interactions (Kipps et al., 2009; McDonald et al., 2003, 2006). This task introduces contextual cues (e.g., prosody, facial movement, and gestures) and additional processing demands (e.g., adequate speed of information processing, selective attention, and social reasoning) that are not taxed when viewing static displays. We only considered part 1, called the emotion evaluation test (EET), which assesses recognition of emotional expression (fearful, surprised, sad, angry, and disgusted). In the EET, speaker demeanor (voice, facial expression, and gesture) together with the social situation indicate the emotional meaning. In some scenes, there is only one actor talking, who is either on the telephone or talking directly to the camera. Other scenes depict two actors and instructions are given to focus on one of them. All scripts are neutral in content and do not lend themselves to any particular emotion. The brief EET comprises a series of 20 short (15–60 s) videotaped vignettes of trained professional actors interacting in everyday situations. After viewing each scene, the test participant is instructed to choose from a forced-choice list the emotion expressed by the focused actor.

#### ToM

##### Reading the mind in the eyes (RMET)

This test (Baron-Cohen et al., 1997) assesses the emotional inference aspect of the ToM (or empathic accuracy). This is a computerized and validated test in which consist of 17 pictures of the eye region of a face. Participants are asked to choose which of four words best describes what the person in each photograph is thinking or feeling.

##### Faux pas test (FPT)

The FPT assesses the emotional and cognitive inference aspects of the ToM. In this task, the participants read stories that may contain a social faux pas (Stone et al., 1998). After each story was read, the subject is asked whether someone said something awkward (in order to identify stories containing a faux pas). Each story was presented in front of the patient in order to decrease working memory load. Performance was scored regarding the adequate identification of the faux pas (hits) and the adequate rejection of those stories which did not contain a faux pas (rejects). The score was 1 point for each faux pas correctly identified (maximum: 10), or non-faux pas correctly rejected (maximum: 10). A total score was computed (out of 20 total points) by adding the number of hits and rejects. When a faux pas was correctly identified, subjects were also asked 2 additional questions to measure intentionality—that is, recognizing that the person committing the faux pas was unaware that they had said something inappropriate (maximum 10)—and emotional attribution, in which participants should recognize that the person hearing the faux pas might have felt hurt or insulted (maximum 10).

#### Empathy

##### Empathy for pain task (EPT)

The EPT evaluates the empathy in the context of intentional and accidental harms. The task consists of 25 animated situations involving two individuals that are presented successively (Decety et al., 2011). The three following kinds of situations were depicted: intentional pain in which one person (passive performer) is in a painful situation caused intentionally by another (active performer), e.g., stepping purposely on someone's toe (pain caused by other); accidental pain where one person is in a painful situation accidentally caused by another; and control or neutral situations (e.g., one person receiving a flower given by another).

Importantly, the faces of the protagonists are not visible and there was no emotional reaction visible to the participants. We measured the ratings and reaction times (RTs) to situation comprehension (e.g., “press the button as soon as you understand the situation”). In addition, we assessed 7 questions about the following aspects of the scenarios: intentionality, e.g., the accidental or deliberate nature of the action; emphatic concern (how sad you feel for the victim); degree of discomfort (for the victim); harmful behavior (how bad was the purpose of the perpetrator); the valence behavior of the active perpetrator (how much positive emotion he/she felt in performing the action); the correctness of the action (moral judgment); and finally punishment (how much penalty this action deserves). Each question was answered using a computer-based visual analogue scale giving 7 different ratings by trial. Accuracy, RTs and ratings were measured.

Interpersonal Reactivity Index (IRI) (Davis, 1983). The IRI is a 28-item self-report questionnaire that separately measures both the cognitive and affective components of empathy. The instrument contains four scales: Perspective Taking (PT), Empathic Concern (EC), Fantasy (F), and Personal Distress (PD).

#### Moral judgment

##### Moral judgment task

Following the protocol reported elsewhere (Young et al., 2010), we presented participants with 24 scenarios. The four variations of each scenario followed a 2 × 2 design: (1) the protagonists either harmed another person (negative outcome) or did no harm (neutral outcome); (2) the protagonists either believed that they would cause harm (negative intent) or believed that they would cause no harm (neutral intent). Each possible belief was true for one outcome and false for the other outcome. The agent held true beliefs in the all-neutral and all-negative conditions and false beliefs in the accidental harm and attempted harm conditions. The participants saw one version of each scenario. In total, eight possible versions of the 24 scenarios with six trials of each of the four conditions were presented. The stimuli were presented in a pseudorandom order and the conditions were counterbalanced across participants. Each participant read six stories in each of the four conditions. After reading each story, the participants were asked to rate the scenario on a Likert-scale ranging from totally permissible (7) to totally forbidden (1).

#### Social norms knowledge

##### Social norms questionnaire (SNQ)

The SNQ questionnaire consisting of 20 yes–no questions was used (Rankin et al., 2009). The participants were asked to determine whether a behavior would be appropriate in the presence of an acquaintance (not a close friend or family member) according to the mainstream culture. Two scores were derived. The break score was defined as the total number of errors made in the direction of breaking a social norm, and the over-adhere score was defined as the total number of errors made in the direction of over adherence to a perceived social norm.

#### Self-monitoring behavior in social settings

##### Revised self-monitoring scale (RSMS)

The RSMS is a 13-item instrument and assesses the tendency to regulate one's behavior to present a particular self in a social context (Lennox and Wolfe, 1984). The scale involves two styles of self-monitoring behavior: the ability to modify self-presentation (e.g., “in social situations, I have the ability to alter my behavior if I feel that something else is called for”) and the sensitivity to the expressive behavior of others (e.g., “I am often able to read people's true emotions correctly through their eyes”). The participants responded using a 6-point Likert-scale. The ratings ranged from 0 = “strongly disagree” to 5 = “strongly agree.”
